# *Arthrobacter pokkalii* sp nov, a Novel Plant Associated Actinobacterium with Plant Beneficial Properties, Isolated from Saline Tolerant Pokkali Rice, Kerala, India

**DOI:** 10.1371/journal.pone.0150322

**Published:** 2016-03-10

**Authors:** Ramya Krishnan, Rahul Ravikumar Menon, Naoto Tanaka, Hans-Jürgen Busse, Srinivasan Krishnamurthi, Natarajan Rameshkumar

**Affiliations:** 1 Biotechnology Department, National Institute for Interdisciplinary Science and Technology (CSIR), Thiruvananthapuram, 695 019, Kerala, India; 2 NODAI Culture Collection Center, Tokyo University of Agriculture, 1-1-1 Sakuragaoka, Setagaya, Tokyo, 156–8502, Japan; 3 Institute of Microbiology, Veterinary University Vienna, A-1210, Vienna, Austria; 4 Microbial Type Culture Collection & Gene Bank (MTCC), CSIR-Institute of Microbial Technology, Sec-39A, Chandigarh, 160036, India; Yeungnam University, REPUBLIC OF KOREA

## Abstract

A novel yellow colony-forming bacterium, strain P3B162^T^ was isolated from the pokkali rice rhizosphere from Kerala, India, as part of a project study aimed at isolating plant growth beneficial rhizobacteria from saline tolerant pokkali rice and functionally evaluate their abilities to promote plant growth under saline conditions. The novel strain P3B162^T^ possesses plant growth beneficial traits such as positive growth on 1-aminocyclopropane-1-carboxylic acid (ACC), production of indole acetic acid (IAA) and siderophore. In addition, it also showed important phenotypic characters such as ability to form biofilm and utilization of various components of plant root exudates (sugars, amino acids and organic acids), clearly indicating its lifestyle as a plant rhizosphere associated bacterium. Taxonomically, the novel strain P3B162^T^ was affiliated to the genus *Arthrobacter* based on the collective results of phenotypic, genotypic and chemotaxonomic analyses. Moreover, molecular analysis using 16S rRNA gene showed *Arthrobacter globiformis* NBRC 12137^T^, *Arthrobacter pascens* DSM 20545^T^ and *Arthrobacter liuii* DSXY973^T^ as the closely related phylogenetic neighbours, showing more than 98% 16S rRNA similarity values, whereas the *recA* gene analysis displayed *Arthrobacter liuii* JCM 19864^T^ as the nearest neighbour with 94.7% sequence similarity and only 91.7% to *Arthrobacter globiformis* LMG 3813^T^ and 88.7% to *Arthrobacter pascens* LMG 16255^T^. However, the DNA-DNA hybridization values between strain P3B162^T^, *Arthrobacter globiformis* LMG 3813^T^, *Arthrobacter pascens* LMG 16255^T^ and *Arthrobacter liuii* JCM 19864^T^ was below 50%. In addition, the novel strain P3B162^T^ can be distinguished from its closely related type strains by several phenotypic characters such as colony pigment, tolerance to NaCl, motility, reduction of nitrate, hydrolysis of DNA, acid from sucrose, cell wall sugars and cell wall peptidoglycan structure. In conclusion, the combined results of this study support the classification of strain P3B162^T^ as a novel *Arthrobacter* species and we propose *Arthrobacter pokkalii* sp.nov.as its name. The type strain is P3B162^T^ (= KCTC 29498^T^ = MTCC 12358^T^).

## Introduction

The actinobacterial genus *Arthrobacter* [1, 2] including the type species *Arthrobacter globiformis*, is a member of the family *Micrococcaceae*. Phenotypically, almost all species of this genus were reported to share characters such as obligate requirement of oxygen for their growth, exhibiting Gram-positive reaction on Gram staining, negative acidification of glucose, non-motility and to undergo morphological changes from rod shaped cells to sphere shaped cells (cocci) during their growth cycle [3]. Chemotaxonomically, the species of this genus are characterised by peptidoglycan type A3α or A4α as defined by Schleifer and Kandler, [4]. The vast majority of *Arthrobacter* species with peptidoglycan type A3α show a quinone system with MK-9(H2) predominating but species with MK-8(H_2_) have been reported as well (summarized by Busse et al. [3]). The majority of the species sharing peptidoglycan type A4α contains exclusively unsaturated menaquinones (MK-8, MK-9, and/or MK-10) but two species of this peptidoglycan type have been shown to contain MK-8(H_2_) as the major menaquinone [3]. Almost all species of this genus contain cellular fatty acids, majorly dominated by anteiso-C_15:0_, iso-C_15:0_, anteiso-C_17:0_, and iso-C_16:0_ [3]. Recently, the genus *Arthrobacter* has been dissected into eleven *Arthrobacter*-groups mainly based on the 16S rRNA gene sequence phylogeny or high 16S rRNA similarities, and by group specific chemotaxonomic traits [3] and the genus *Arthrobacter sensu stricto* was restricted to *Arthrobacter globiformis*, *Arthrobacter pascens*, *Arthrobacter oryzae* and *Arthrobacter humicola*. Other groups defined were *‘Arthrobacter aurescens-*group’, ‘*Arthrobacter oxydans*-group’, ***‘****Arthrobacter protophormiae-*group’, ‘*Arthrobacter sulfureus*-group’, ‘*Arthrobacter citreus*-group’, ‘*Arthrobacter agilis*-group’, ‘*Arthrobacter psychrolactophilus*-group’, ‘*Arthrobacter pigmenti*-group’, ‘*Arthrobacter albus*/*cumminsii-*group’ and ‘*Sinomonas*-group’.

The genus *Arthrobacter* represents one of the most divergent heterotrophic bacterial groups of actinobacteria, because of their metabolic versatility they are reported to exist in a diverse range of environments like soils, plants, freshwater, clinical specimens and marine habitats [3, 5, 6]. Numerous studies have revealed the association of diverse strains of *Arthrobacter* with different plants by both culture-dependent and -independent methods [7–11]. More importantly it was found in higher portions in plants which are grown in saline, drought, polluted and low nutrient agricultural soils, where they were found to be beneficial to the plants by protecting them from abiotic stress and improve plant nutrition, health and yield [12–15]. Because of these plant beneficial properties, they were considered to be an important member among the plant growth promoting rhizobacteria that are present in the rhizosphere microflora [9, 13, 16]. Despite their importance towards plant health under stressful conditions, the knowledge about the association of *Arthrobacter* with different crop plants that are growing in stressful conditions and its functional significance is far from adequate. The discovery of a new *Arthrobacter* species from these crop plants as in this case pokkali rice, a highly saline tolerant rice variety and dealing with its plant probiotic properties is therefore still significant.

Till date, there are more than 70 described species with validly published names in the genus *Arthrobacter* that were isolated from various sources [17]. Notably recovered from plant rhizosphere are *Arthrobacter cupressi* [18], *A*. *siccitolerans* [19], *A*. *bambusae* [6], *A*. *gyeryongensis* [20], *A*. *oryzae* [21] and *A*. *humicola* [21]. However, none of these above studies provided any evidence that they possess plant growth beneficial properties even though they are isolated from different plant rhizosphere.

During an investigation on the taxonomy and functional characterization of rhizosphere bacteria of a saline tolerant pokkali rice, a strain designated as P3B162^T^ was isolated. The current study describes the isolation, plant beneficial functions and taxonomic position of this novel *Arthrobacter* strain P3B162^T^ using a polyphasic approach as recommended by Schumann et al. [22]. Based upon the collective results generated in this study, a new species of the genus *Arthrobacter*, *Arthrobacter pokkalii* sp. nov., is proposed.

## Materials and Methods

### Ethics statement

There is no requirement for any authority permit to collect the plant rhizosphere samples. The rhizosphere samples were collected from various rice fields with the knowledge and prior permission from the respective owners of the field. This study doesn’t involve any endangered or protected plant species.

### Isolation of bacterial strain P3B162^T^

Strain P3B162^T^ was isolated from the rhizosphere soil of a hybrid pokkali rice variety VTL-6 collected from Alappuzha in Kerala, India. A standard serial dilution procedure was followed in isolating the bacterial strain P3B162^T^. Briefly, two grams of soil attaching to the root portion was collected carefully and mixed with 20ml of sterile 0.85% (w/v) NaCl solution. The resulting rhizosphere soil suspension was then vortexed vigorously for 10-30min and was allowed to stand still for 5min. A fivefold serial dilutions were done with the above rhizosphere soil suspension and 0.1ml aliquots of each dilution was then spread plated onto 1/1000 dilutions of Luria Bertani (LB) agar medium (Himedia, India), pH 5.5 (maintained with 0.1N HCl). These plates were then incubated for 1–2 weeks. Strain P3B162^T^ was regularly subcultured on full strength LB agar medium at 28°C for 3–4 days. For storage purposes, the strain was kept at 4°C as active plates for 2–3 weeks as short-term storage and as 10% glycerol suspensions stored in deep freezer at −80°C as long-term storage. The following reference strains were included in this study; *Arthrobacter globiformis* LMG 3813^T^, *A*. *pascens* LMG 16255^T^, *A*. *liuii* JCM 19864^T^, *A*. *humicola* DSM 25587^T^, *A*. *oryzae* DSM 25586^T^ and *A*. *cupressi* DSM 24664^T^.

### Phylogenetic analysis using 16S rRNA and *rec*A genes

The genomic DNA was extracted from overnight grown bacterial cells using the DNA extraction kit (QIAamp DNA Mini Kit; Qiagen) as per the protocol mentioned in the manufacturer’s instruction manual. The 16S rRNA gene amplification with primers 27F and 1492R [23] were performed as reported before [24]. The *recA* gene was amplified using the primers GPRA-UF2 (5′- GGSAAGGGSKCNGTNATGCG-3′) and GPRA-UR2 (5′-CCTTSCCCTGSCCNARYT-3′) [25] using PCR program of 5 min at 95°C, 33 cycles of 1 min at 95 C, 1 min at 56°C and 1 min at 72°C, and 10 min at 72°C as final extension step. The PCR product size of 1500bp (16S rRNA) and 800bp (*recA*) was gel eluted using Gel Extraction kit (Qiagen) following the manufacturer’s protocol and bi-directionally sequenced using the ABI Prism BIGDye Terminator v3.1 cycle sequencing ready reaction kit (Applied Biosystems). For 16S rRNA gene sequencing, the 16S rRNA internal primers as mentioned by Rameshkumar *et al*. [24] were used and for *recA* gene sequencing GPRA-UF2 and GPRA-UR2 primers were used. The Applied Biosystems 3500 DNA Genetic Analyzer was used for sequencing purposes. The quality gene sequences thus obtained were matched against the reference 16S rRNA and *rec*A gene sequences that are available in the National Center for Biotechnology Information (NCBI) database using BLASTn search tool. Using Eztaxon [26], nearest phylogenetic neighbours and its 16S rRNA gene sequence similarity values were determined. The sequences were aligned using CLUSTAL X1.8 [27] and corrected by manual inspection. Phylogenetic trees were reconstructed using three different tree making algorithms; neighbour-joining, maximum parsimony and maximum-likelihood methods with Kimura two-state parameter model analyses as described before [28] except that the program MEGA version 5 was used [29]. Using NCBI ORF finder, the *recA* nucleotide sequences were translated to amino acid sequences based on the putative open reading frames and NCBI BLASTp search tool was used to find identical protein sequences that are present in the protein database.

### Genomic fingerprinting

#### Repetitive extragenic palindromic PCR (rep-PCR)

Whole DNA fingerprinting was performed by using four (rep-PCR) primers; REP1R-I & REP2-I, enterobacterial repetitive intergenic consensus (ERIC1R & ERIC2), BOXA1R, and (GTG)_5_ as described [30,31,24] using PCR program of 5 min at 95°C, 35 cycles of 1 min at 95°C, 1 min at 40°C and 8 min at 65°C, and then a final extension step of 10 min at 72°C. The experiment was repeated three independent times to make sure the fingerprints generated are reproducible.

### GC content and DNA-DNA hybridization

The procedure of Marmur [32] was followed to isolate high molecular weight genomic DNA for GC content analysis. By determining the mid-point (*T*_m_) of the thermal denaturation curve of the given DNA [33] and by using the equation of Owen & Hill [34], the G+C base composition was calculated. DNA-DNA hybridization (DDH) was conducted using fluorometric direct binding method [35] and the hybridization experiments were performed in micro dilution wells. For labelling the DNA as probes, photoprobe biotin from Vector Laboratories, Inc., was used. The temperature used for DNA-DNA hybridization was 53°C.

### Chemotaxonomy

For analysis of cellular fatty acids, cells were grown on Tryptic Soy Agar (TSA-Himedia) for 2 days at 30°C and cells were harvested at similar physiological age. The fatty acids were extracted and determined as mentioned before [28, 36] except MIDI Sherlock TSBA6 (version of the database) was used for identification of the fatty acid methyl esters. The peptidoglycan structure and whole cell wall sugars analyses were determined according to the protocol described by Schumann [37] at DSMZ using service facility provided for bacterial identification. For polar lipids and quinone analyses stationary phase grown cells were used and 3.3xPYE broth which contains 1.0% peptone from casein, and 1.0% yeast extract, (pH 7.2) was used to raise the biomass. The extraction and analyses of quinones and polar lipids were done as reported recently [38, 39] and for quinone analysis the HPLC equipment reported by Stolz et al. [40] was applied.

### Physiological and biochemical analysis

Bacterial cell shape and motility were confirmed using Leica DM 2000 microscope using cells grown in LB broth at 30°C and observations were made from 12 to 72 h. The Gram reaction, catalase, and oxidase tests were done accordingly [28]. Growth in various bacteriological media was tested on nutrient agar, trypticase soya agar, R2A and MacConkey agar. The anaerobic growth was tested on LB agar medium supplemented with 25mM each of glucose and citrate and on LB agar medium supplemented with 10mM potassium nitrate. These inoculated plates were incubated in a jar maintained under anaerobic conditions by using anaerobic catalysts (Himedia). Growth at various temperatures (4, 18, 30, 37 and 42°C) and sodium chloride concentrations (0, 1, 2, 4, 6, 8 and 10%, w/v) was tested in Luria broth containing tryptone 10g/L, and yeast 5g/L (HiMedia). Growth at different pH range between pH 5.0 to 11.0 were checked with the following buffers: 0.1M citrate buffer for pH 5; 0.1M MES for pH 5.5 and 6; 0.1M Na_2_CO_3_/0.1M NaHCO_3_ for pH 8 and 9; 0.05M NaHCO_3_ /0.1M NaOH for pH 10 and 11 and LB broth medium was used as the basal medium. Indole production, nitrate reduction, urease and Methyl red and Voges proskauer (MR-VP) tests were done as mentioned earlier [41]. Hydrolysis of casein, chitin, carboxymethyl cellulose, pectin, starch, and xylan were determined as reported earlier [28] except the basal medium used was LB agar medium in all tests. Hydrolysis of esculin and DNA, arginine dihydrolase, lysine decarboxylase and utilisation of citrate using simmon’s citrate agar were determined as before [28] Esterase activity was determined using tributyrin agar basal medium (Himedia) added with 1% tributyrin (Sigma). After week incubations at 28°C, positive and negative results were recorded. HiCarbohydrate kit (Himedia) was used to check acid production from various carbohydrates. For inoculation, the culture suspensions were made in 0.85% NaCl (w/v), and the results were observed after 48 h at 28°C. Utilization of D-glucose, L-arabinose, maltose, trehalose, D-mannitol, xylose, cellobiose, D-fructose, sucrose, sorbitol, malate and citrate as sole carbon sources were determined in minimal medium broth (MM); K_2_HPO_4_-0.8g/L, KH_2_PO_4_-0.2g/L, MgSO_4_.7H_2_O-0.2g/L, CaCl_2._2H_2_O-0.2g/L, NaCl-5g/L, NH_4_Cl-1g/L and with 0.1% w/v of the respective carbon sources. Utilization of various amino acids such as alanine, arginine, asparagine, aspartate, cysteine, glutamate, glutamine, glycine, histidine, isoleucine, leucine, lysine, methionine, phenylalanine, proline, putrescine, serine, threonine, tryptophan, tyrosine and valine as sole nitrogen sources were determined in MM broth with glucose-1g/L as sole carbon source and NH_4_Cl-1g/L was replaced with respective (1g/L) amino acids. Results of utilization tests were recorded as positive or negative based on the growth of the cells after week incubations at 30°C. Antibiotic sensitivity tests were determined as reported earlier [28] except the tests were performed on LB agar medium. The antibiotic discs with known concentrations were used (μg/disc; Himedia): ampicillin (10), carbenicillin (100), chloramphenicol (30), ciprofloxacin (5),clindamycin (2), doxycycline hydrochloride (30), erythromycin (15), gentamicin (10), kanamycin (30), linezolid (30), methicillin (5), nalidixic acid(30), ofloxacin (5), oxacillin (1) and penicillin G (10 units), polymixin B (300 units), rifampicin (5), streptomycin (10), tetracycline (30), vancomycin (30).

### Plant growth functions

The utilisation of 1-aminocyclopropane carboxylic acid (ACC) as sole nitrogen source was determined using the method as reported by Penrose & Glick [42] with slight modification. The isolate was grown in a minimal agar medium consisting of 5g/L each of glucose and sucrose, K_2_HPO_4_-0.8g/L, KH_2_PO_4_-0.2g/L, MgSO_4_.7H_2_O-0.2g/L, CaCl_2._2H_2_O-0.2g/L, NaCl-5g/L, agar-18g/L and 3mM ACC as sole nitrogen source. After a week incubation, visible growth on the medium considered as positive for ACC utilisation [43]. To check Indole Acetic acid (IAA) production, the isolate was grown in a minimal broth medium consisting of 5g/L of sucrose, K_2_HPO_4_-0.8g/L, KH_2_PO_4_-0.2g/L, MgSO_4_.7H_2_O-0.2g/L, CaCl_2._2H_2_O-0.2g/L, NaCl-5g/L and supplemented with 1g/L tryptophan. After an incubation period of 3 days at 30°C, the quantification of IAA was determined using the Salkowski assay [44]. Ability to form biofilm was determined using the method reported by Christensen et al. [45] with the modifications as mentioned by Sarkar and Chakraborty, [46] the tests were performed in McCarthy tubes (HiMedia). Siderophore production was determined using the method as mentioned before [47]. Ability to grow in semi-solid nitrogen free medium was tested by growing the isolate in minimal medium devoid of any nitrogen source: 5g/L of sucrose, K_2_HPO_4_-0.8g/L, KH_2_PO_4_-0.2g/L, MgSO_4_.7H_2_O-0.2g/L, CaCl_2._2H_2_O-0.2g/L, NaCl-5g/L and agar-2g/L, incubated for 5 days at 30°C. A visible pellicle formation on the medium was considered as positive. Presence of nitrogen fixing gene was checked by PCR amplification of *nifH* gene using primers 19F (5’-GCIWTYTAYGGIAARGGIGG-3’) and 407R (5’-AAICCRCCRCAIACIACRTC-3’) as reported by Rameshkumar et al. [24]. Ability of the isolate to grow under drought stress was analysed as cited by Marasco et al. [48] except that the isolate was grown in 10% and 20% Polyethylene Glycol (PEG) prepared in Luria Bertani broth medium.

### Nucleotide sequence accession number

The GenBank/EMBL/DDBJ accession numbers for the 16S rRNA gene of strain P3B162^T^ (KM507333) and *recA* gene of strain P3B162^T^ (KP202159), *Arthrobacter humicola* DSM 25587^T^ (KP202160), *Arthrobacter oryzae* DSM 25586^T^ (KP202161), *Arthrobacter cupressi* DSM 24664^T^ (KP939172) and *Arthrobacter liuii* JCM 19864^T^ (KP939173) respectively.

## Results and Discussion

**Fig 1:**
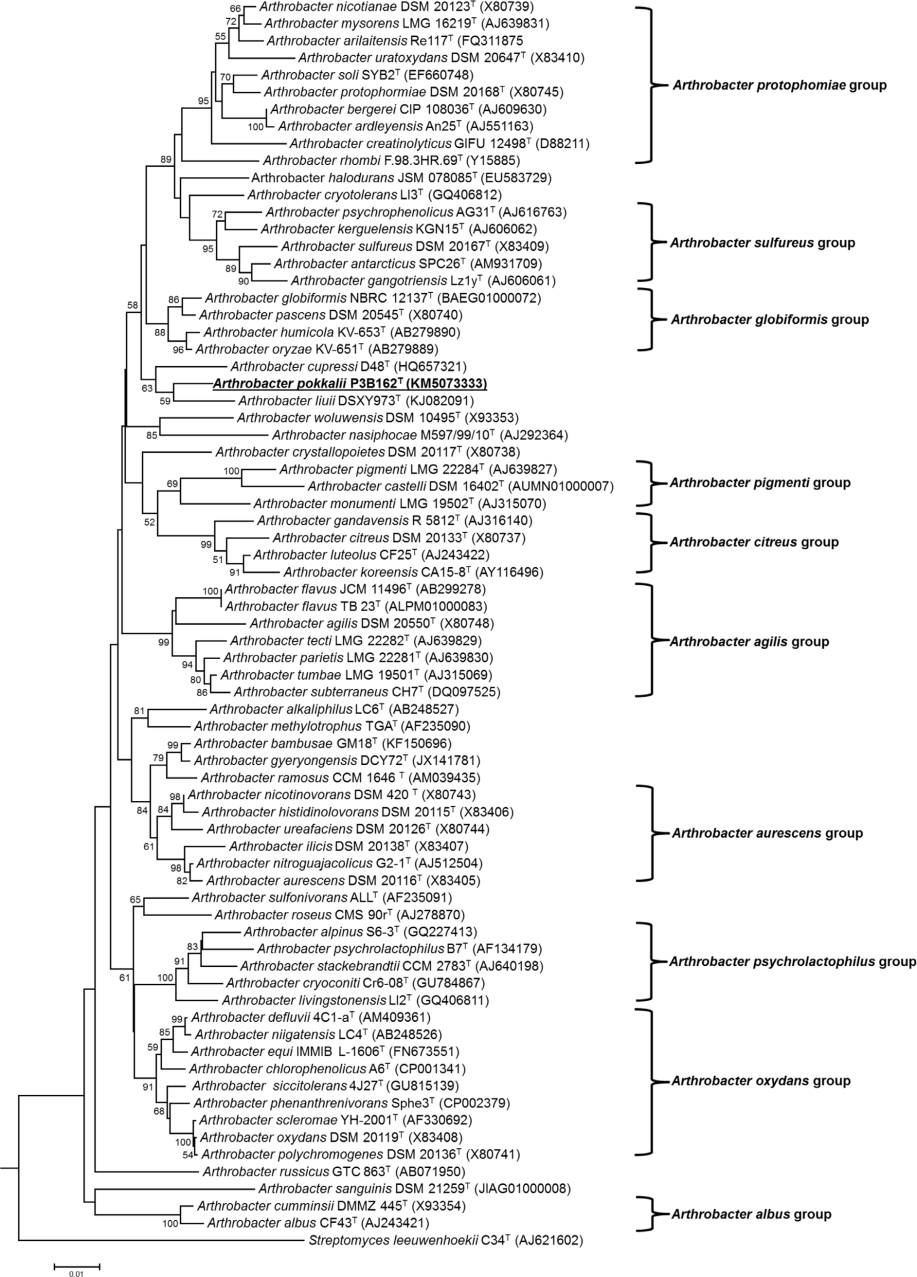
Maximum-likelihood tree constructed using 16S rRNA nucleotide sequences, displaying the phylogenetic position of strain P3B162^T^ within the genus *Arthrobacter*. Bar, 0.01 substitutions per nucleotide position. The sequence of *Streptomyces leeuwenhoekii* C34^T^ served as an out group.

The 16S rRNA phylogenetic analysis, comprising 1424bp revealed that strain P3B162^T^ belongs to the genus *Arthrobacter* (Fig 1), displaying higher pairwise 16S rRNA gene sequence similarities to the type strains of two *Arthrobacter* species which are representatives of the genus *Arthrobacter sensu stricto* [3]; *Arthrobacter globiformis* NBRC 12137^T^ (98.17%), and *A*. *pascens* DSM 20545^T^ (98.10%). Followed by *A*. *liuii* DSXY973^T^ with 98.02% sequence similarity, very recently described species so far has not been assigned to any *Arthrobacter* group. No 16S rRNA similarity values higher than 98% were found with other type strains of established *Arthrobacter species*. It is worth to mention here that other *Arthrobacter* species namely *A*. *phenthrenivorans* Sphe3^T^, *A*. *defluvii* 4C1-a^T^, *A*. *niigatensis* LC4^T^, representatives of *Arthrobacter oxydans*-group and *A*. *tecti* LMG 22282^T^, a representative of *Arthrobacter agilis-*group shared relatively high 16S rRNA similarity values between 97.5 to 97.8% with strain P3B162^T^. However, all these 16S rRNA similarity values are lesser than 98.5%, the mean value considered to be the threshold for the existence of a novel species within a genus [49]. Altogether these informations clearly provided the evidence for assignment of strain P3B162^T^ as a novel species in the genus *Arthrobacter*. However such observations should be carefully validated by a polyphasic taxonomy approach which involves characterization based on phenotypic and genotypic methods [22, 50]. In 16S rRNA maximum-likelihood phylogenetic tree the novel strain P3B162^T^ occupied a distinct phylogenetic position moving away from *A*. *globiformis* NBRC 12137^T^ and *A*. *pascens* DSM 20545^T^ but clustering stably with *A*. *liuii* DSXY973^T^ (Fig 1). When other treeing algorithms were applied (maximum parsimony, neighbour-joining) again *A*. *liuii* DSXY973^T^ was phylogenetically identified as the next relative of strain P3B162^T^ (S1 and S2 Figs). However, the phylogenetic analyses do not provide reliable evidence that P3B162^T^ is related to any other species of the genus *Arthrobacter*. Based on the high 16S rRNA similarity and considering the phylogenetic positioning of P3B162^T^ the following type strains were used for further taxonomic comparison: *Arthrobacter globiformis* LMG 3813^T^, *A*. *pascens* LMG 16255^T^, *A*. *liuii* JCM 19864^T^, *A*. *humicola* DSM 25587^T^, *A*. *oryzae* DSM 25586^T^ and *A*. *cupressi* DSM 24664^T^.

The results of 16S rRNA gene sequence comparisons and its corresponding phylogeny did not provide information on relationship to any *Arthrobacter* group as defined by Busse et al. [3]. Therefore, we analysed the housekeeping gene encoding recombinase *A* (*recA*) of strain P3B162^T^ and its related reference *Arthrobacter* species. This gene is considered to be of greater taxonomic resolution than the 16S rRNA gene, when describing and differentiating closely related strains up to species level [22, 50–53]. A partial (770) bp of *recA* sequence of strain P3B162^T^ was obtained after PCR amplification and sequencing. The resulting sequence was compared with *recA* sequences available from the databases and determined in the course of this study. The result also identified *A*. *liuii* JCM 19864^T^ as the next relative of P3B162^T^ with highest *recA* sequence similarity (94.7%). Other *Arthrobacter* species showed *recA* sequence similarities of 88.7–91.7% with strain P3B162^T^ (Table 1). Except *A*. *globiformis*, species assigned to *Arthrobacter sensu stricto* showed relatively low similarity values (88.7–89.4%) whereas similarity values with species of *Arthrobacter oxydans-*group were significantly higher (91.1–91.7%) (Table 1). Interestingly, in the corresponding amino acid sequence of RecA (trimmed to 159 amino acids according to the shortest sequence of an *Arthrobacter* type species accessible) strain P3B162^T^ shared highest similarity (99.3%) with four species of *Arthrobacter oxydans-*group namely *A*. *oxydans* ATCC 14358^T^, *A*. *phenanthrenivorans* Sphe3^T^, *A*. *polychromogenes* ATCC 15216^T^ and *A*. *siccitolerans* 4J27^T^ *a*nd only 98.7% with *A*. *liuii* JCM 19864^T^, 98.1% with each, *A*. *globiformis* LMG3813^T^, *A*. *pascens* LMG 16255^T^, *A*. *oryzae* DSM 25586^T^, *A*. *cupressi* DSM 24664^T^ and *A*. *chlorophenolicus* A6^T^ (S1 Table). Likewise, very high amino acid sequence similarities were also observed among several type strains of *Arthrobacter* species notably 100% amino acid sequence similarity between four species of *Arthrobacter oxydans-*group namely *A*. *oxydans* ATCC 14358^T^, *A*. *phenanthrenivorans* Sphe3^T^, *A*. *polychromogenes* ATCC 15216^T^ and *A*. *siccitolerans* 4J27^T^, between *A*. *pascens* ATCC 13346^T^ and *A*. *oryzae* DSM 25586^T^ and between *A*. *globiformis* ATCC 8010^T^ and *A*. *chlorophenolicus* A6^T^ (S1 Table), even though these type strains of *Arthrobacter* species share very low similarity at the nucleotide level among each other (Table 1). However, when longer stretches (212–250 amino acids) of the same gene of selected species were compared, the amino acid similarity values between aforementioned species decreased significantly. For instance, in this comparison *A*. *phenanthrenivorans* Sphe3^T^ shared only 97.2% similarity with *A*. *pascens* ATCC 13346^T^ and *A*. *chlorophenolicus* A6^T^ shared 97.2% similarity with *A*. *globiformis* LMG 3813^T^ instead of 98.7 and 100% similarity observed in the shorter amino acid sequences, respectively well reflecting the relationships indicated from 16S rRNA phylogeny and its assignment to respective *Arthrobacter* groups. From these observations it is obvious that at least some relationships indicated by high RecA similarities in the short amino acid sequences are misleading and no taxonomic conclusions should be drawn without good support from other data. It is also clear that the trimmed corresponding *recA* nucleotide sequences provide better taxonomic resolution to closely related *Arthrobacter* strains than its amino acid sequences. This observation is most likely due to the degeneracy of the genetic code, where in many cases a mutation at the third position of a nucleotide triplet does not affect the translated amino acid. Hence, in pairwise sequences comparisons, the amino acid sequence similarity value was always higher than that of its corresponding DNA sequences (Table 1 and S1 Table). Similar findings were obtained by other researchers when using other protein coding genes for similar phylogenetic studies; *HSP60* gene [54, 55], *gyrB* gene [56]. Since only rather short *recA* sequences (450 nucleotides) were accessible we did not carry out phylogenetic calculations. However, both *recA* and RecA data identified *A*. *liuii* JCM 19864^T^ as the next relative of P3B162^T^. The results from *recA* gene sequence comparisons also supported the status of P3B162^T^ as a novel species because the similarity in this gene sequence was lower (94.7%) than that found between the very closely related *Arthrobacter* species; *A*. *polychromogenes* ATCC 12516^T^ and *A*. *oxydans* ATCC 14358^T^ (97.10%).

**Table 1:** *recA* gene sequence similarity values between strain P3B162^T^ and its related *Arthrobacter* type strains.

Strain	Sequence similarity (%)										
	1	2	3	4	5	6	7	8	9	10	11
1. P3B162^T^	100										
2. *A*. *liuii* JCM 19864^T^	94.7	100									
3. *A*. *globiformis* LMG 3813^T^	91.7	90.8	100								
4. *A*. *pascens* LMG 16255^T^	88.7	89.5	91.8	100							
5. *A*. *humicola* DSM 25587^T^	89.2	88.7	88.7	88.9	100						
6. *A*. *oryzae* DSM 25586^T^	89.4	89.8	89.1	90.2	92.5	100					
7. *A*. *cupressi* DSM 24664^T^	91.6	89.4	90.9	88.9	89.1	89.8	100				
8. *A*. *oxydans* ATCC 14358^T^	91.1	89.4	90.8	90.6	89.6	89.4	89.6	100			
9. *A*. *phenanthrenivoarns Sphe3*^*T*^	91.1	88.9	88.9	88.9	88.1	89.8	88.7	92.7	100		
10. *A*. *chlorophenolics* A6^T^	91.6	91.4	92.5	88.0	88.9	88.3	89.6	89.2	88.1	100	
11. *A*. *polychromogenes* ATCC 15216^T^	91.6	90.7	91.7	91.3	89.8	90.5	91.4	97.1	93.6	89.4	100
12. *A*. *siccitolerans* 4J27^T^	91.7	91.1	89.9	89.2	90.6	92.6	90.4	91.0	91.5	90.2	91.0

In addition to the phylogenetic analysis using 16S rRNA and *recA* genes, we used rapid DNA typing methods based on whole genome to check whether the strain P3B162^T^ is genetically different from its closest type strains. For this purpose, repetitive extragenic palindromic genomic fingerprinting methods using different set of primers was adapted and the results clearly proved that the DNA fingerprints of strain P3B162^T^ was evidently different from the DNA fingerprinting patterns of closely related *Arthrobacter* species [*A*. *globiformis* LMG 3813^T^, *A*. *pascens* LMG16255^T^, *A*. *liuii* JCM 19864^T^, *A*. *humicola* DSM 25587^T^, *A*. *oryzae* DSM 25586^T^ and *A*. *cupressi* DSM 24664^T^ (S3 Fig)]. Though different genomic fingerprint do not necessarily identify different species but the results support the conclusion derived from the sequence analyses using two genes encoding 16S rRNA and *recA*, respectively that strain P3B162^T^ may likely to represent a novel species in the genus *Arthrobacter*.

In order to validate distinct species status for strain P3B162^T^, the DNA-DNA hybridization between the strain P3B162^T^ and its closely related phylogenetic neighbours sharing more than 98% 16S rRNA similarity; *A*. *globiformis* LMG 3813^T^, *A*. *pascens* LMG 16255^T^ and *A*. *liuii* JCM 19864^T^ were chosen and performed. The results showed that, at the DNA-DNA level, the relatedness between P3B162^T^ and its closest phylogenetic neighbours were found to be 40.8% with *A*. *liuii* JCM 19864^T^, 31.9% with *A*. *globiformis* LMG 3813^T^, and 41.5% with *A*. *pascens* LMG 16255^T^. This result was further well supported by the *recA* sequence analysis, where the novel strain P3B162^T^ shared low level of gene sequence similarities (<95%) with its closely related species of the genus *Arthrobacter* (Table 1), clearly indicating the suitability of using *recA* gene as a substitute in describing new *Arthrobacter* species before considering DNA–DNA hybridization experiments. However, the results of DNA relatedness undoubtedly pointed towards the fact that strain P3B162^T^ does not belong to any of its closest *Arthrobacter* species when considering the cut-off value of 70% DNA–DNA relatedness for the definition of species is considered [57]. These data clearly indicate that strain P3B162^T^ should represent a novel species status in the genus *Arthrobacter*.

Nevertheless, description of novel species involves merely not based on the genomic differences, but also should be validated through a polyphasic approach which includes both phenotypic and chemotaxonomic data [53].

Results of phenotypic data validated the phylogenetic analysis results by placing the strain P3B162^T^ in the genus *Arthrobacter*. This assignment of novel strain P3B162^T^ to the genus is based on the phenotypic characters which are shared by almost all species of the genus *Arthrobacter* such as non-motility, strict requirement of oxygen for growth, positive in Gram staining, displaying a rod to coccus cell morphology in its growth cycle and no production of acid from glucose [3]. Colonies of the novel isolate grown on LB agar medium are yellow pigmented (S4 Fig) and other characters including biochemical and physiological features of strain P3B162^T^ were detailed and presented in its species description. The identified phenotypic characters which distinguish strain P3B162^T^ from it’s phylogenetically closely related *Arthrobacter* species are mentioned in Table 2.

**Table 2:** Identified useful phenotypic characters distinguishing strain P3B162^T^ from its closely related *Arthrobacter* type strains as inferred from 16S rRNA gene sequence phylogeny and high 16S rRNA similarity value.

Phenotypic characters	1	2	3	4	5	6	7
Colony pigment^a^	Yellow	-	-	-	-	-	-
Motility	-	-	-	-	+	+	-
Growth in 8% NaCl	+	-	-	-	-	-	-
Nitrate reduction	+	+	-	-	-	+	-
**Hydrolysis:**							
DNA	+	-	-	-	w	-	-
Esculin	+	+	+	-	+	-	-
Tributyrin	+	+	-	-	w	-	+
Utilisation of L-arabinose	+	+	+	+	-	-	+
**HICarbohydrate kit**							
**Utilisation of:**							
ONPG	+	-	-	-	+	+	+
Malonate	+	-	-	-	-	-	-
**Acid Production:**							
Fructose	+	-	+	+	-	-	-
Rhamnose	-	-	+	+	-	-	-
Sucrose	+	-	+	+	-	-	-
Cell wall sugar	Galactose Rhamnose mannose	Galactose mannose ribose rhamnose	Galactose glucose	Galactose glucose	Galactose rhamnose	Galactose glucose	Galactose Glucose mannose ribose
G+C content (mol%)	64	67.6	62–65	63.7	67	67	65
Peptidoglycan interpeptide bridge	A3α-Lys-Ser-Thr-Ala	A3α-Lys-Ser-Thr-Ala	A3α-Lys-Ala_3_	A3α-Lys-Ala_2_	A3α-Lys-Ala_>2_	A3α-Lys-Ala_>2_	A3α-Lys-Ala_2_

Strains: 1, P3B162^T^; 2, *A*.*liuii* JCM 19864^T^; 3, *Arthrobacter globiformis* LMG 3813^T^; 4, *A*. *pascens* LMG 16255^T^; 5, *A*. *humicola* DSM 25587^T^; 6, *A*. *oryzae* DSM 25586^T^; 7, *A*.*cupressi* DSM 24664^T^_._ All data are from this study except data for motility, cell wall sugars, G+C content and peptidoglycan interpeptide bridge for *Arthrobacter globiformis* LMG 3813^T^; *A*. *pascens* LMG 16255^T^; *A*. *humicola* DSM 25587^T^; *A*. *oryzae* DSM 25586^T^*; A*.*liuii* JCM 19864^T^ and *A*. *cupressi* DSM 24664^T^ were taken from previously published work [3,21,58,18]

+, Positive; -, Negative; w, weak reaction; ND, No Data available

^a^ Colony morphology of novel strain P3B162^T^ grown on Luria agar medium (Himedia) incubated for 72h at 30°C.

Next, we evaluated the strain P3B162^T^ for its plant growth beneficial traits in plate assays. The results revealed that strain P3B162^T^ able to produce plant growth phytohormone, Indole acetic acid (IAA) (106.7±6.48 μg/ml) from L-tryptophan, which is a well-known plant growth regulator, showed positive growth on 1-aminocyclopropane-1-carboxylic acid (ACC) (S5A Fig), indicating its ability to utilize ACC, a precursor of ethylene biosynthesis thereby protects the plants from the harmful effects of stress ethylene produced during abiotic stress where many studies have shown ACC utilizing rhizobacteria known to help plants under abiotic stress conditions [59, 60], positive for siderophore production (S5B Fig) and can form biofilm on the walls of the glass tube (S5C Fig), an important trait used by many rhizosphere bacteria for efficient plant root colonization [61, 62]. The ability of the isolate to grow in 10% and 20% PEG solutions clearly indicated its possibility to perform its function under drought conditions as well [48, 63]. However the novel strain P3B162^T^ found negative for growth in semisolid nitrogen free medium and for nitrogen fixation gene (*nifH*) amplification. These results altogether indicate the possible role of strain P3B162^T^ towards plant growth promotion as previous reports state that rhizosphere bacteria which possess these plant growth beneficial activities have positive influence on the plant growth [64–66].

We also identified a number of phenotypic characters which might contribute to the successful adaptability of this novel strain P3B162^T^ towards its existence in plant rhizosphere. It grows in pH 5.5, tolerates up to 8% NaCl, clearly depicting the acid saline nature of the site from where this novel isolate P3B162^T^ was isolated. It can grow both in oligotrophic as well as in high nutrient conditions clearly indicating its metabolic versatility of the novel isolate P3B162^T^ towards surviving under the fluctuating nutrient levels in the rhizosphere environments [67]. It utilises humic acid which is a major component of the soil [68], utilises certain plant derived components which are secreted as root exudates such as sucrose [69], citric acid, DL-malic acid [70], L-arabinose, D-cellobiose, citric acid, D-dextrose, D-fructose, D-galactose, D-dextrose, maltose, xylose [71], tryptophan and putrescine [72–74]. Additionally, it utilises components which are majorly present in root exudates when the plant is under stress conditions; proline and mannitol [75, 76]. It produces enzymes such as pectinase (S5D Fig), proteases, lipase and amylase which might be useful in hydrolysing the plant polymers [77]. These results altogether gives a positive indication that this novel strain P3B162^T^ possesses typical characters which can be selective advantage when it is residing in plant rhizosphere, were similar findings identified in other plant growth promoting rhizobacteria [78, 79]. Therefore, it will be interesting to explore further to know more in detail about the beneficial interactions between the novel strain and its host plant, the saline tolerant pokkali rice plant and how it protects the plant from salinity stress and is being explored through ongoing plant inoculation studies.

**Fig 2:**
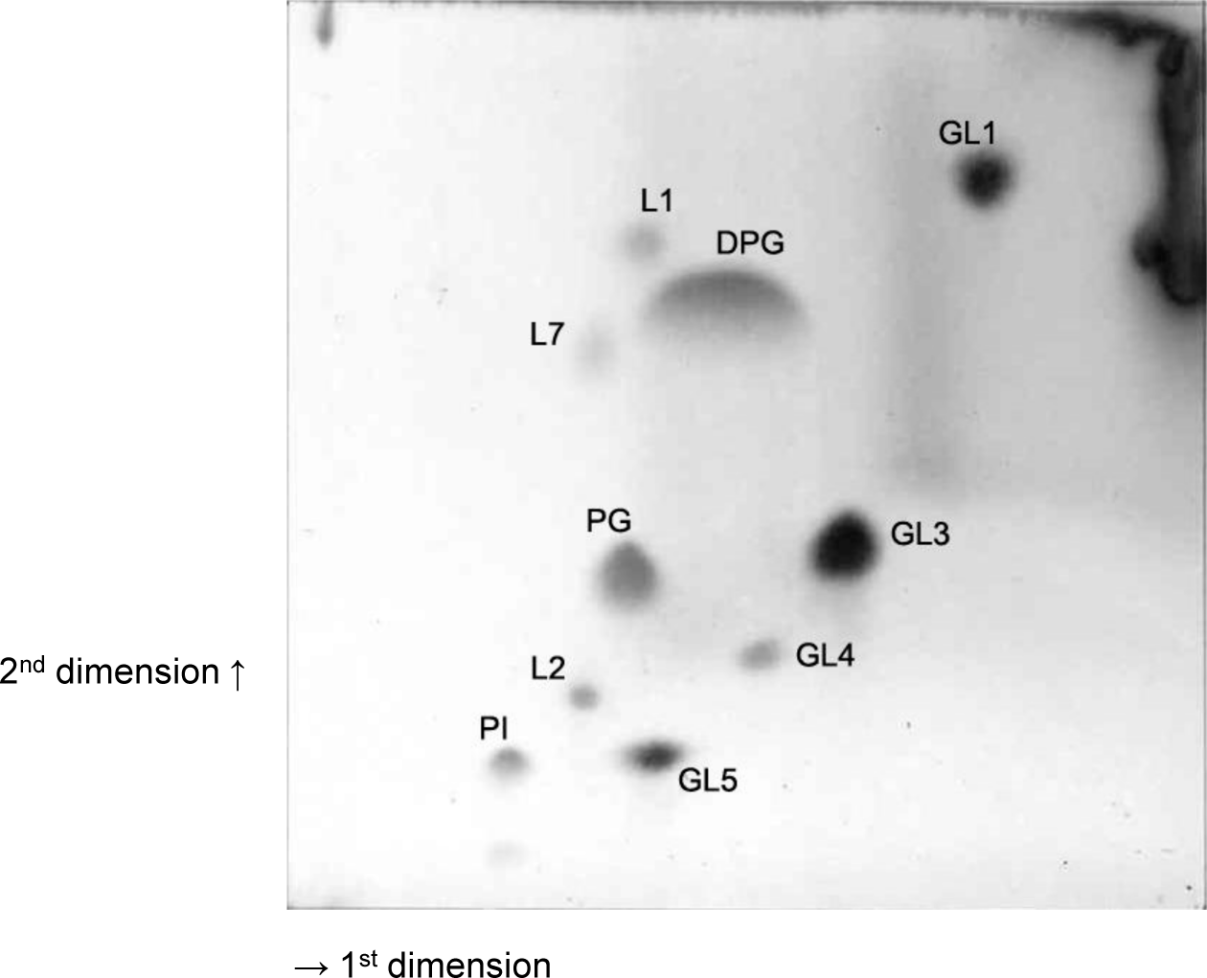
Polar lipid profile of strain P3B162^T^, after two dimensional TLC and detection with molybdatophosphoric acid, DPG, diphosphatidylgylcerol; PG, phosphatidylglycerol; PI, phosphatidylinositol; GL1, monogalactosyldiacylglycerol; GL3, dimannosylglyceride; GL5, trimannosyldiacylglycerol; GL4, unidentified glycolipid; L1, L2, L7, unidentified lipids.

Chemotaxonomically strain P3B162^T^ contained fatty acids majorly dominated by anteiso-C_15:0_ (53.98%), anteiso-C_17:0_ (14.62%), iso- C_16:0_ (8.64%), iso-C_15:0_ (8.06%), C_16:0_ (7.14%), a fatty acids profile which is similar to that of representatives of the genus *Arthrobacter*. The fatty acids profile of strain P3B162^T^ and its phylogenetically closely related *Arthrobacter* species are mentioned in Table 3. The quinone system of P3B162^T^ contained predominantly menaquinones, 88.1% MK-9(H_2_), 9.1% MK-8(H_2_) and 2.8% MK-10(H_2_) which is in line with the quinone system of numerous *Arthrobacter* species. The polar lipid profile (Fig 2) showed the major lipids as diphosphatidylglycerol, phosphatidylglycerol, dimannosylglyceride, digalactosyldiacylglycerol and trimannosyldiacylglycerol. In addition, minor to moderate amounts of phosphatidylinositol, an unidentified glycolipid (GL4) and three lipids (L1, L2 and L7) only detectable after total lipid staining. This polar lipid profile is similar to the profile of *Arthrobacter globiformis*, *Arthrobacter pascens*, *Arthrobacter humicola*, (*Arthrobacter sensu stricto*), *Arthrobacter polychromogenes* (‘*Arthrobacter oxydans-*group), *Arthrobacter histidinolovorans* (*Arthrobacter aurescens-*group), *A*. *liuii* and *A*. *cupressi and* clearly distinguishes from profiles of other *Arthrobacter* species (Busse, unpublished results; [58]). In detail, strain P3B162^T^ could be distinguished from *A*. *globiformis* NBRC 12137^T^ based on the presence of glycolipid GL4, lipids L1, L2, L7 and higher amount of trimannosyldiacylglycerol (identified in the image of the polar lipid profile of *A*. *globiformis* NBRC 12137^T^ based on its chromatographic motility) in its polar lipid profile as given in [58]. Furthermore, the presence of phospholipid PL1 in the profile of *A*. *globiformis* NBRC 12137^T^ was not detectable in P3B162^T^. Polar lipid profiles of strain P3B162^T^ and *A*. *liuii* DSXY973^T^ were more similar to each other especially based on the presence of GL4 designated GL3 in *A*. *liuii* DSXY973^T^ [58]. However, also these two strains could be distinguished from each other based on the presence of lipids L1, L2 and L7 in the polar lipid profile strain P3B162^T^.

**Table 3:** Whole cell fatty acids composition of strain P3B162^T^ and its phylogenetically closest neighbors. Taxa: 1, P3B162^T^; 2, *Arthrobacter liuii* JCM 19864^T^; 3, *A*. *globiformis* LMG 3813^T^; 4, *A*. *pascens* LMG 16255^T^; 5, *A*. *humicola* DSM 25587^T^; 6, *A*. *oryzae* DSM 25586^T^; 7, *A*. *cupressi* DSM 24664^T^. All data were obtained in this study. Only fatty acids amounting 0.5% or higher are shown. t, traces (<0.5%).

Fatty acids (%)	1	2	3	4	5	6	7
**Saturated**							
C_14:0_	1.38	2.21	2.69	0.61	0.76	1.5	t
C_16:0_	7.14	3.80	6.13	3.78	3.9	6.51	1.90
**Branched-chain**							
C_14:0_ iso	1.52	3.46	2.35	0.5	t	t	1.49
C_15:0_ iso	8.06	5.77	4.75	5.79	4.22	1.84	2.54
C_15:0_ anteiso	53.98	62.30	62.97	50.38	63.87	64.15	66.33
C_16:0_ iso	8.64	8.85	8.8	5.93	4.76	3.28	9.36
C_17:0_ iso	1.35	t	0.58	1.74	0.79	t	0.55
C_17:0_ anteiso	14.62	8.68	10.39	21.28	17.16	18.03	15.85

The cell wall peptidoglycan analysis showed that the cell wall of strain P3B162^T^ contains A3α peptidoglycan type [4] with L-Lys–L-Ser–L-Thr–L-Ala as interpeptide bridge, which is similar to the cell wall peptidoglycan structure of *A*. *liuii* JCM 19864^T^. However, this peptidoglycan structure clearly distinguishes P3B162^T^ from *Arthrobacter sensu stricto* and places it in the vicinity of ‘*Arthrobacter oxydans*-group’ as defined by Busse et al. [3]. The cell wall peptidoglycan structure plays a major place in the *Arthrobacter* taxonomy as many *Arthrobacter* species have been grouped based on their cell wall peptidoglycan structure Busse et al. [3]. Considering the peptidoglycan structure which is in line to that of *A*. *oxydans* group, the phenotypic characters that are differentiating P3B162^T^ from the *Arthrobacter oxydans* group were given in S2 Table. The cell-wall sugars of strain P3162^T^ was composed of galactose, rhamnose and mannose an important character that differentiates strain P3B162^T^ from its closest neighbours (Table 2 and S2 Table).

### Taxonomic conclusion

In summary, the phenotypic, chemotaxonomic and phylogenetic data obtained in this study showed that strain P3B162^T^ belongs to the genus *Arthrobacter*. The results of phylogenetic analysis using 16S rRNA and *recA*, genomic fingerprinting using different DNA typing methods, DNA-DNA hybridization, and phenotypic data distinguished the strain P3B162^T^ from its closest neighbours *A*. *globiformis* LMG 3813^T^, *A*. *pascens* LMG16255^T^, *A*. *liuii* JCM 19864^T^, *A*. *humicola* DSM 25587^T^, *A*. *oryzae* DSM 25586^T^and *A*. *cupressi* DSM 24664^T^. Therefore, strain P3B162^T^ is classified as a novel species in the genus *Arthrobacter*, for which the name *Arthrobacter pokkalii* sp. nov. is proposed.

### *Arthrobacter pokkalii* sp.nov. Species Description

*Arthrobacter* pokkalii (pok.ka'li.i. N.L. gen. n. pokkalii of pokkali (a variety of rice).

Cells are rod shaped during log phase and as cocci in stationary/older phase during its growth cycle, Gram staining positive, non-motile, requires oxygen for its growth and no growth in anaerobic conditions, catalase-positive, and oxidase-negative. Colonies on LB agar medium are yellow pigmented with circular, slightly convex and colony size reaches approximately 1.0 to 2.0 mm in diameter after 3 days of incubation at 30°C. Growth occurs in different bacteriological medias; R2A, nutrient agar, TSA agar medium but no growth was observed in MacConkey agar. The growth was observed between temperatures 18°C to 37°C (optimum at 28–30°C). At temperature 4°C and 42°C no growth was observed. Growth occurs between pH 5.5 to 8 (optimum at pH 7) and with NaCl concentrations 0 to 8% (w/v) (optimum at 0.5 to 1%). Casein, esculin, DNA, lipid, starch, and pectin are hydrolysed whereas negative hydrolysis for urea, chitin, carboxymethyl cellulose, xylan and gelatin. Negative reaction for arginine dihydrolase, lysine decarboxylase and MR-VP tests. Indole is not produced and nitrate is reduced to nitrite. In the HiCarbohydrate kit, fructose and sucrose showed positive acid production whereas adonitol, L-arabinose, D-arabinose, arabitol, cellobiose, dextrose, dulcitol, erythritol, galactose, glycerol, inositol, inulin, lactose, maltose, mannitol, mannose, melibiose, α methyl D-glucoside, α methyl D-mannoside, melezitose, raffinose, rhamnose, salicin, sodium gluconate, sorbitol, sorbose, trehalose, xylitol and xylose showed negative acid production. ONPG and malonate are utilised but citrate is not utilised. L-arabinose, D-cellobiose, citric acid, D-dextrose, D-fructose, D-galactose, humic acid, DL-malic acid, maltose, D-mannitol, sucrose and xylose are utilised as sole carbon sources but D-sorbitol is not utilised. L-alanine, L-aspartate, L-arginine, L-asparagine, L-cysteine, L-glutamine, L-glutamate, L-glycine, L-histidine, L-isoleucine, L-leucine, L-lysine, L-phenylalanine, L-proline, putrescine, L-serine, L-threonine, L-tryptophan, L-tyrosine and L-valine are utilised as sole nitrogen sources. Senstivity to the following antibiotics (μg/disc): ampicillin (10), carbenicillin (100), chloramphenicol (30), ciprofloxacin (5), clindamycin (2), doxycycline hydrochloride (30), erythromycin (15), gentamicin (10), kanamycin (30), linezolid (30), nalidixic acid (30), ofloxacin (5), oxacillin (1) and penicillin G (10 units), rifampicin (5), streptomycin (10), tetracycline (30), vancomycin (30) and intermediate to methicillin (5) and polymixin B (300 units). It contains fatty acids majorly as anteiso-C_15:0_, anteiso-C_17:0_, iso-C_16:0_, iso-C_15:0_, and C_16:0_. Minor amount of fatty acids with less than 0.2% are C_12:0_, anteiso-C_13:0_, C_17:0_ cyclo, C_17:0_ 3OH, C_18:1_ *ω*9c, C_18:1_ 2OH, anteiso-C_19:0_ and summed feature 3 (C_16:1_*ω*7c/C_16:1_*ω*6c). It contains A3α peptidoglycan type with L-Lys–L-Ser–L-Thr–L-Ala as interpeptide bridge and the cell-wall sugars as galactose, rhamnose and mannose. It contains major menaquinones as MK-9(H_2_) and minor amounts of MK-8(H_2_) and MK-10(H_2_). It contains polar lipids majorly as diphosphatidylglycerol, phosphatidylglycerol, dimannosylglyceride, digalactosyldiacylglycerol and trimannosyldiacylglycerol. In addition, minor amounts of phosphatidylinositol, an unidentified glycolipid (GL4) and three lipids (L1, L2 and L7) only detectable after total lipid staining. Positive for plant growth beneficial traits such as growth on 1-aminocyclopropane carboxylic acid as nitrogen source, produce phytohormone, indole-3-acetic acid and siderophore, can form biofilm and has the ability to grow in 10 and 20% PEG solutions. Negative for growth in semisolid nitrogen free medium and presence of nitrogen fixation gene (*nifH*).

The type strain P3B162^T^ (= KCTC 29498^T^ = MTCC 12358^T^) was isolated from the rhizosphere of saline tolerant pokkali rice of Alappuzha, Kerala, India. The DNA G+C content of strain P3B162^T^ is 64.0 mol%.

## Supporting Information

S1 Fig Neighbour-joining tree based on the nucleotide sequences of 16S rRNA gene, showing the phylogenetic position of strain P3B162^T^ within the genus *Arthrobacter*. Bar, 0.01 substitutions per nucleotide position. The sequence of *Streptomyces leeuwenhoekii* C34^T^ served as an out group.
(TIF)

S2 Fig Maximum parsimony tree based on the nucleotide sequences of 16S rRNA gene, showing the phylogenetic position of strain P3B162^T^ within the genus *Arthrobacter*. Bar, 0.01 substitutions per nucleotide position. The sequence of *Streptomyces leeuwenhoekii* C34^T^ served as an out group.
(TIF)

S3 Fig PCR-based genomic fingerprinting using following primers; BOX, (GTG)_5_, ERIC and REP performed between strain P3B162^T^ and its phylogenetically closest *Arthrobacter* type strains. (TIF)

S4 Fig Colony morphology of strain P3B162^T^ grown on LB agar medium after 72 h at 30°C. (TIF)

S5 Fig Plant growth beneficial traits of strain P3B162^T^ by invitro plate assays. (a) Plate showing growth of strain P3B162^T^ after an incubation of 7 days on minimal agar medium supplemented with 3mM ACC. (b) Plate showing positive siderophore production by forming an orange halo zone around the grown cells after 7 days of incubation. (c) A glass tube assay showing biofilm formation of strain P3B162^T^ after an incubation of 24 h at 30°C. Adherence of the cells was detected by staining with crystal violet that is shown as a ring. (d) Plate assay showing positive for pectinase by formation of a halo zone around the grown cells in pectin amended minimal medium on treatment with iodine after an incubation of 7 days.
(TIF)

S1 Table *recA* amino acid sequence similarity values between strain P3B162^T^ and its related *Arthrobacter* type strains. (DOCX)

S2 Table Phenotypic and genotypic characters differentiating strain P3B162^T^ from *Arthrobacter* strains having similar peptidoglycan interpeptide bridge of L-Lys–L-Ser–L-Thr–L-Ala. (DOCX)
