# Supplementary figures and images for: Arthrobacter pokkalii sp nov, a Novel Plant Associated Actinobacterium with Plant Beneficial Properties, Isolated from Saline Tolerant Pokkali Rice, Kerala, India
Source: PLoS One. 2016 Mar 10;11(3):e0150322. doi: 10.1371/journal.pone.0150322 (PMC4786123; doi:10.1371/journal.pone.0150322)

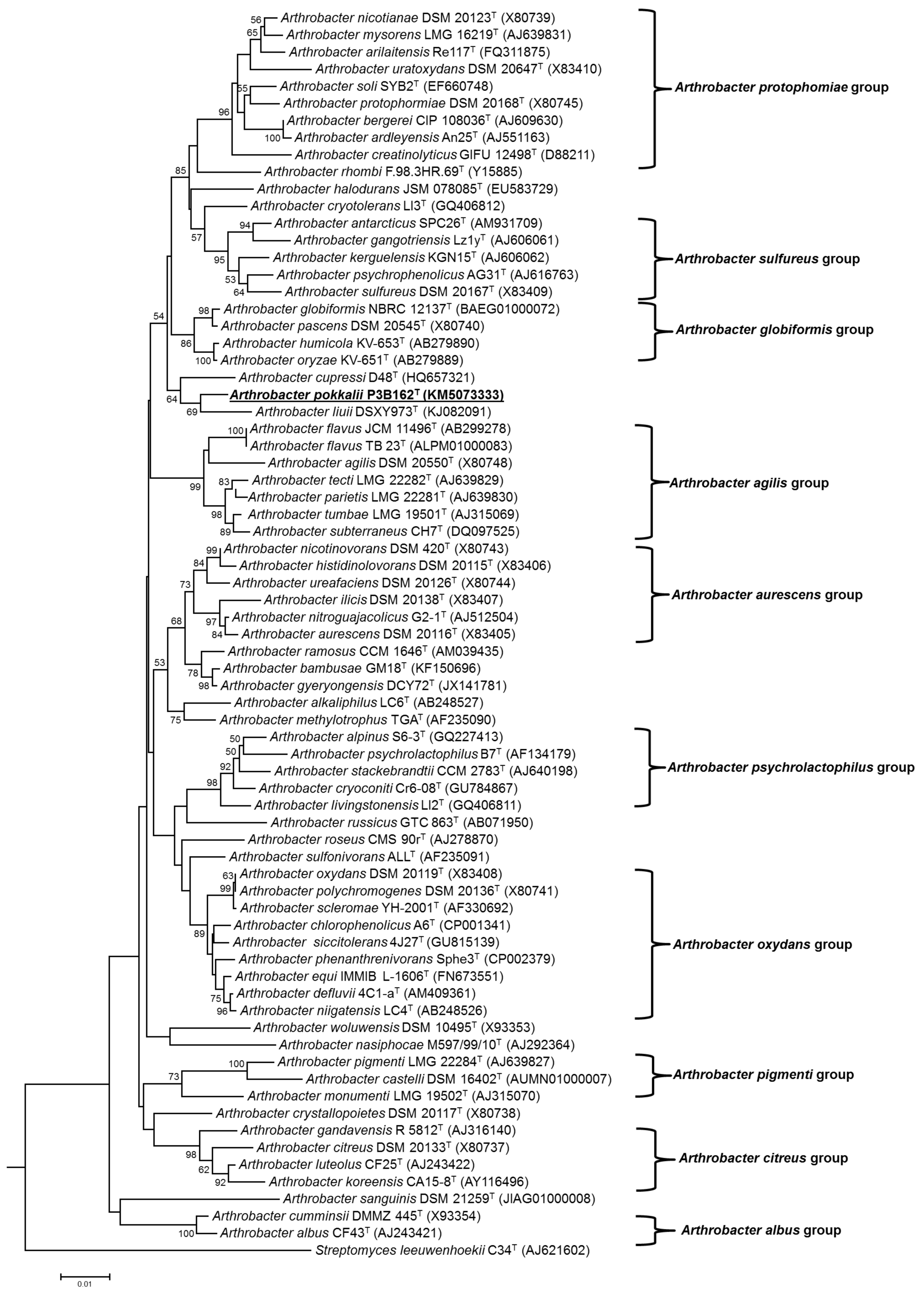

Supplement: S1 Fig — Bar, 0.01 substitutions per nucleotide position. The sequence of Streptomyces leeuwenhoekii C34T served as an out group. (TIF) [file pone.0150322.s001.tif]

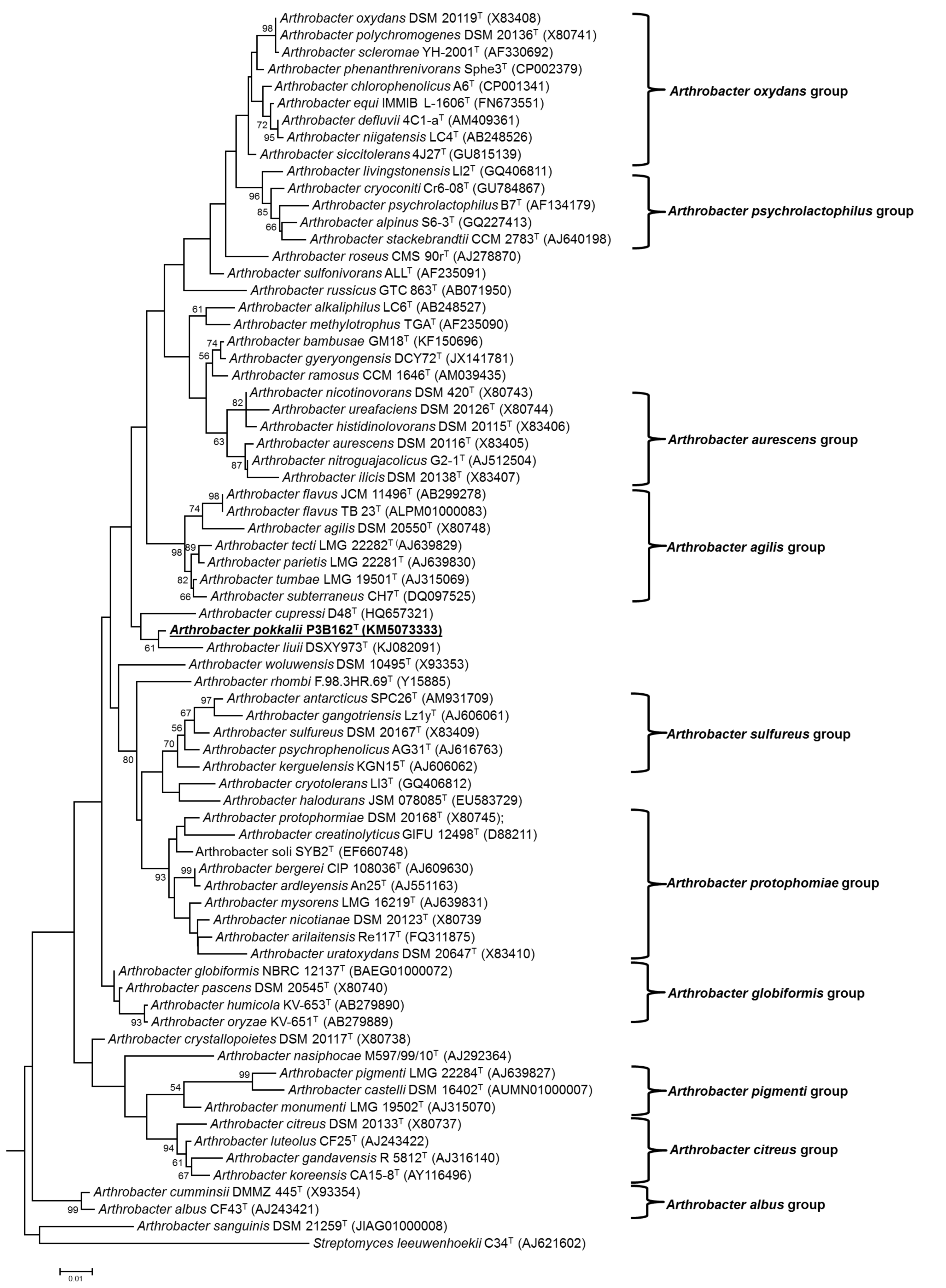

Supplement: S2 Fig — Bar, 0.01 substitutions per nucleotide position. The sequence of Streptomyces leeuwenhoekii C34T served as an out group. (TIF) [file pone.0150322.s002.tif]

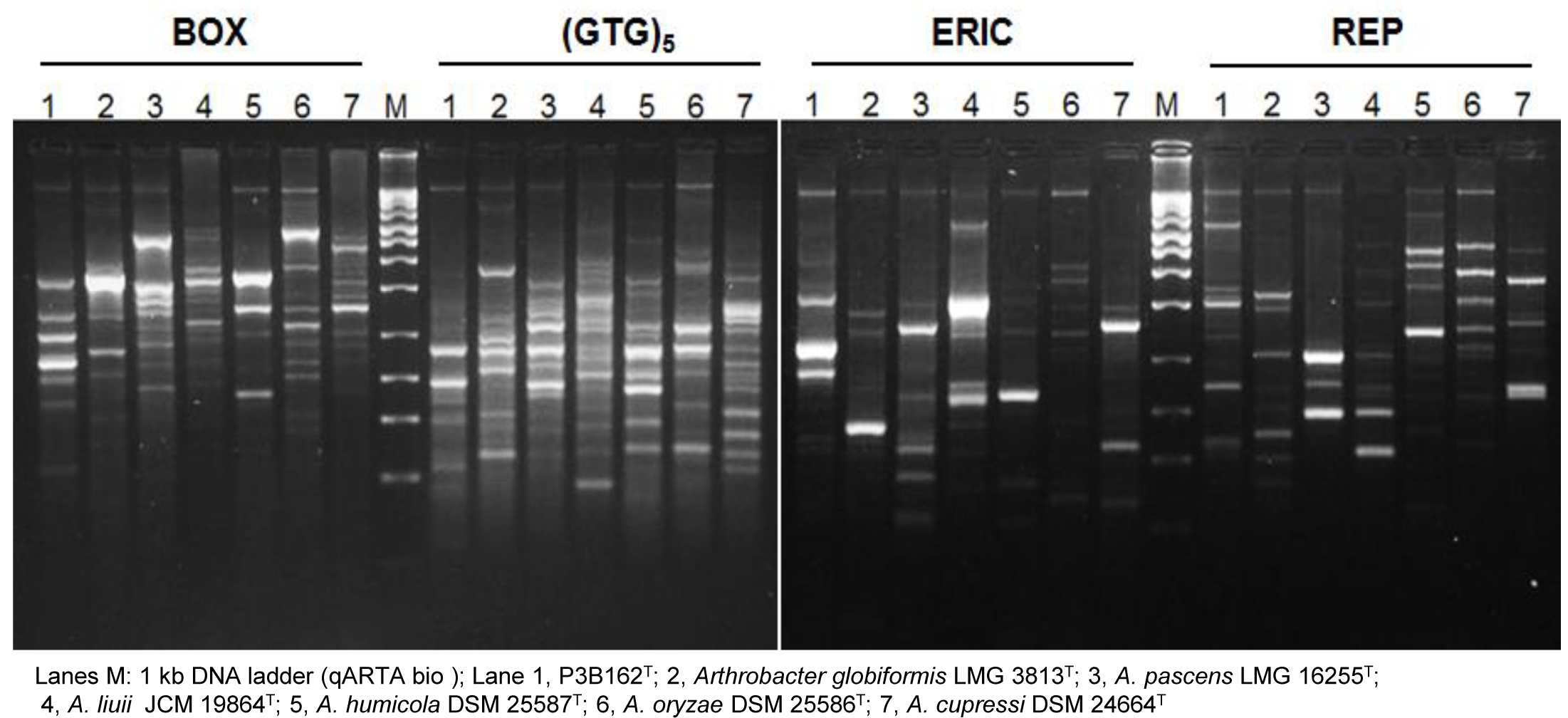

Supplement: S3 Fig — (TIF) [file pone.0150322.s003.tif]

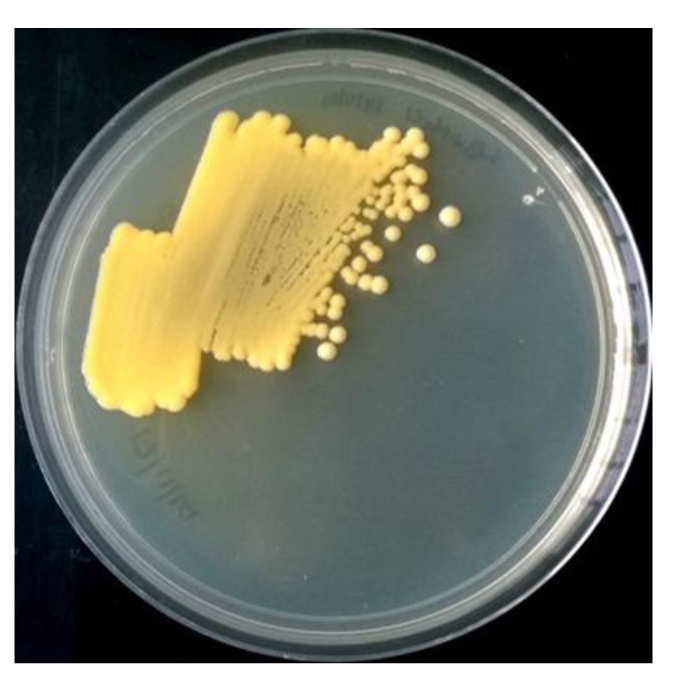

Supplement: S4 Fig — (TIF) [file pone.0150322.s004.tif]

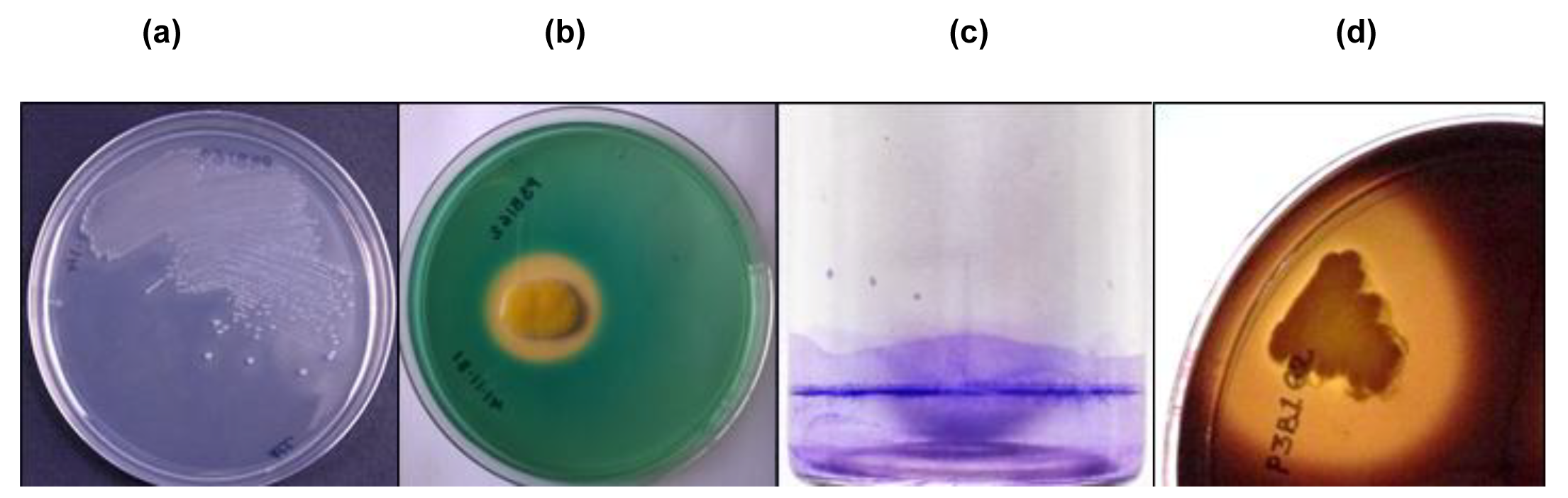

Supplement: S5 Fig — (a) Plate showing growth of strain P3B162T after an incubation of 7 days on minimal agar medium supplemented with 3mM ACC. (b) Plate showing positive siderophore production by forming an orange halo zone around the grown cells after 7 days of incubation. (c) A glass tube assay showing biofilm formation of strain P3B162T after an incubation of 24 h at 30°C. Adherence of the cells was detected by staining with crystal violet that is shown as a ring. (d) Plate assay showing positive for pectinase by formation of a halo zone around the grown cells in pectin amended minimal medium on treatment with iodine after an incubation of 7 days. (TIF) [file pone.0150322.s005.tif]
