# Supplementary material for: Arthrobacter pokkalii sp nov, a Novel Plant Associated Actinobacterium with Plant Beneficial Properties, Isolated from Saline Tolerant Pokkali Rice, Kerala, India
Source: PLoS One. 2016 Mar 10;11(3):e0150322. doi: 10.1371/journal.pone.0150322 (PMC4786123; doi:10.1371/journal.pone.0150322)
Supplement: S1 Table — (DOCX) [file pone.0150322.s006.docx]

**S1 Table.** *recA* amino acid sequence similarity values between strain P3B162^T^ and its related *Arthrobacter* type strains.

| **Strain** | **Sequence similarity (%)** | | | | | | | | | | |
| --- | --- | --- | --- | --- | --- | --- | --- | --- | --- | --- | --- |
|  | 1 | 2 | 3 | 4 | 5 | 6 | 7 | 8 | 9 | 10 | 11 |
| 1. P3B162^T^ | 100 |  |  |  |  |  |  |  |  |  |  |
| 1. *A*. *liuii* JCM 19864^T^ | 98.7 | 100 |  |  |  |  |  |  |  |  |  |
| 1. *A*. *globiformis* LMG 3813^T^ | 98.1 | 98.1 | 100 |  |  |  |  |  |  |  |  |
| 1. *A. pascens* LMG 16255^T^ | 98.1 | 96.8 | 97.4 | 100 |  |  |  |  |  |  |  |
| 1. ***A. humicola* DSM 25587^T^** | 96.8 | 95.6 | 96.2 | 98.7 | 100 |  |  |  |  |  |  |
| 1. ***A. oryzae*DSM 25586^T^** | 98.1 | 96.8 | 97.4 | 100 | 98.7 | 100 |  |  |  |  |  |
| 1. *A*. *cupressi* DSM 24664^T^ | 98.1 | 96.8 | 97.4 | 96.2 | 95.0 | 96.2 | 100 |  |  |  |  |
| 1. *A. oxydans* ATCC 14358^T^ | 99.3 | 98.1 | 97.4 | 98.7 | 97.4 | 98.7 | 97.4 | 100 |  |  |  |
| 1. *A. phenanthrenivoarns Sphe3^T^* | 99.3 | 98.1 | 97.4 | 98.7 | 97.4 | 98.7 | 97.4 | 100 | 100 |  |  |
| 1. *A. chlorophenolics* A6^T^ | 98.1 | 98.1 | 100 | 97.4 | 96.2 | 97.4 | 97.4 | 97.4 | 97.4 | 100 |  |
| 1. *A. polychromogenes* ATCC 15216^T^ | 99.3 | 98.1 | 97.4 | 98.7 | 97.4 | 98.7 | 97.4 | 100 | 100 | 97.4 | 100 |
| 1. *A. siccitolerans* 4J27^T^ | 99.3 | 98.1 | 97.4 | 98.7 | 97.4 | 98.7 | 97.4 | 100 | 100 | 97.4 | 100 |
