# Supplementary material for: Arthrobacter pokkalii sp nov, a Novel Plant Associated Actinobacterium with Plant Beneficial Properties, Isolated from Saline Tolerant Pokkali Rice, Kerala, India
Source: PLoS One. 2016 Mar 10;11(3):e0150322. doi: 10.1371/journal.pone.0150322 (PMC4786123; doi:10.1371/journal.pone.0150322)
Supplement: S2 Table — (DOCX) [file pone.0150322.s007.docx]

**S2 Table.** Characteristics differentiating strain P3B162^T^ from *Arthrobacter* strains having similar peptidoglycan interpeptide bridge of L-Lys–L-Ser–L-Thr–L-Ala. Strains: **1**, P3B162^T^; **2**, ***A. phenanthrenivorans* Sphe3^T^=DSM 18606^T^**; **3**, ***A. defluvi* 4C1-a^T^ =DSM 18782^T^*; 4,*** ***A. niigatensis* LC4^T^*; 5,*** *A. chlorophenolicus* DSM 12829^T^; **6**, *A. equi* IMMIB L-1606^T^; ***7,*** *A. oxydans* DSM 20119^T^**; 8, *A. polychromogenes* DSM 120116^T^; 9, *A. alkaliphilus* LC6^T^; 10, *A. siccitolerance* 4J27^T^; +, Positive; -, Negative; w, weak; NA, Data not available.** Data for P3B162^T^are from this study. Data for ***A. phenanthrenivorans* DSM 18606^T^*,*** *A. defluvi* **4C1-a^T^;**  ***A. niigatensis* LC4^T^;** *A. chlorophenolicus* DSM 12829^T^;  *A. equi* IMMIB L-1606^T^ *A. oxydans* DSM 20119^T^; ***A. polychromogenes* DSM 120116^T^;** ***A. alkaliphilus* LC6^T^** and *A. siccitolerance* **4J27^T^** were from previously published works [1-7].

| **Differential characters** | **1** | **2** | **3** | **4** | **5** | **6** | **7** | **8** | **9** | **10** |
| --- | --- | --- | --- | --- | --- | --- | --- | --- | --- | --- |
| 16S rRNA similarity | 100% | 97.82% | 97.75% | 97.54% | 97.33% | 97.29% | 97.26% | 97.26% | 97.26% | 97.23% |
| Isolation source | Rhizosphere | Soil | sewage | Filtration substrate | soil | Veterinary Clinical sample | Tobacco leaves | Air | Filtration substrate | Rhizosphere |
| Motility | - | - | - | - | + | - | - | - | - | - |
| Colony colour | Yellow | Cream to yellow | Creamy white | Light gray to yellow | Pearl gray | Cream | Pearl gray to yellow | Blue | Light yellow | Cream |
| Growth at temperature | 18-37 ⁰C | 4-37⁰C | 5-37⁰C | 5-40⁰C | 3-37⁰C | 10-35⁰C | NA | 10-37⁰C | 5-40⁰C | 15-35⁰C |
| pH | 5.5-8 | 6.5-8.5 | 6.0-10.0 | 6.0-11.0 | NA | 6.0-9.0 | 5.0-9.0 | 6.0-11.0 | 6.0-11.0 | 5.0-9.0 |
| Growth at 5% NaCl | + | NA | + | + | + | - | + | + | + | - |
| Growth at 10% NaCl | - | NA | - | - | - | - | + | - | - | - |
| Reduction of nitrate | + | + | + | + | - | - | + | + | - | - |
| **Hydrolysis of:** |  |  |  |  |  |  |  |  |  |  |
| gelatin | - | - | - | + | + | + | + | + | - | - |
| Starch | + | + | + | NA | - | + | - | + | NA | NA |
| **Utilization of:** |  |  |  |  |  |  |  |  |  |  |
| Maltose | + | NA | + | - | + | + | + | + | + | + |
| Sucrose | + | NA | + | + | + | + | - | + | - | + |
| D-xylose | + | NA | + | W | + | + | + | + | - | + |
| sorbitol | - | NA | - | NA | + | + | + | + | NA | + |
| DNA G+C content | 64 mol% | 65.7 mol% | 64.4 mol% | 70.8 mol% | 65.1 mol% | 67 mol% | 63.1 mol% | 62.9 mol% | 69 mol% | 65.3 mol% |
| Cell wall sugars | Galactose, rhamnose,  mannose | NA | Galactose, glucose, rhamnose | NA | NA | NA | Galactose, glucose | NA | NA | Galactose, glucose, mannose, ribose, rhamnose |

**References**

1. Busse HJ, Wieser M, Buczolits S. Genus III. *Arthrobacter* In. Whitman WB, Parte A, Goodfellow M, Kampfer P, Busse HJ, et al. editors. Bergey's Manual of Systematic Bacteriology. 2012. 5: pp-578-625.
2. Santacruz-calvo L, González-lópez J, Manzanera M. *Arthrobacter siccitolerans* sp. nov., a highly desiccation-tolerant, xeroprotectant-producing strain isolated from dry soil.int. J Syst Evol Microbiol. 2013; 63: 4174-4180
3. Kallimanis A, Kavakiotis K, Perrisynakis A, Sproer C, Pukall R, Drainas C, Koukkou AI. *Arthrobacter phenanthrenivorans* sp. nov., to accommodate the phenanthrene-degrading bacterium *Arthrobacter* sp. strain Sphe3. Int J Syst Evol Microbiol. 2009; 59: 275-279.
4. Kim KK, Lee KC, Oh HM, Kim MJ, Eorn MK, Lee JS. *Arthrobacter defluvi* sp. nov., 4-chlorophenol-degrading bacteria isolated from sewage. Int J Syst Evol Microbiol. 2008; 58(8):1916-1921.
5. Yassin AF, Sproer C, Siering C, Hupfer H, Schumann P.  *Arthrobacter equi* sp. nov. isolated from veterinary clinical material. Int. J . Syst. Evol. Microbiol. 2011; 61: 2089-2094
6. Westerberg K, Elvang AM, Stackebrandt E, Jansson JK. *Arthrobacter chlorophenolicus* sp. nov., a new species capable of degrading high concentrations of 4-chlorophenol. Int J Syst Evol Microbiol. 2000; 50: 2083-2092.
7. Ding L, Hirose T, Yokota A. Four novel *Arthrobacter* species isolated from filtration substrate. Int J Syst Evol Microbiol. 2009; 59: 856-862.
